# Bioavailability of Triprolidine as a Single Agent or in Combination With Pseudoephedrine: A Randomized, Open‐Label Crossover Study in Healthy Volunteers

**DOI:** 10.1002/cpdd.777

**Published:** 2020-03-04

**Authors:** Salvatore Febbraro, Tim Shea, Ana Santos Cravo

**Affiliations:** ^1^ PRA Health Sciences Reading UK; ^2^ Reckitt Benckiser Health LLC Parsippany New Jersey USA; ^3^ Reckitt Benckiser Healthcare Ltd Slough UK

**Keywords:** triprolidine hydrochloride, pharmacokinetics, bioavailability, cold, common, antihistamines, anticholinergic activity

## Abstract

Antihistamines have been in clinical use for more than 70 years to treat allergic and nonallergic symptoms including relief from cold and flu symptoms. Despite their widespread use, pharmacokinetic (PK) data are sparse for older, first‐generation antihistamines. This phase 1 single‐center open‐label, randomized, single‐dose, 3‐way crossover trial evaluated the PK profiles of 2 doses of film‐coated triprolidine caplets (2.5 and 5 mg) compared with a reference combination tablet (triprolidine 2.5 mg + pseudoephedrine 60 mg) in 24 healthy adults. Blood samples were collected predose and at specified intervals across a 24‐hour period after administration, and triprolidine was quantified using liquid chromatography‐tandem mass spectrometry. Maximum plasma concentration of triprolidine for the 2.5 mg and dose‐normalized 5 mg single‐agent tablets were comparable (8.4 versus 7.1 ng/mL, respectively) and higher for the combination tablet (9.5 ng/mL). PK parameters, including time to maximum plasma concentration (∼1.5 hours) and elimination half‐life (∼4 hours), were comparable between the 3 treatment arms. The safety profile of this sedating antihistamine was as expected; however, adverse effects were reported in a markedly higher proportion of women than men. There were no significant sex differences in any of the measured PK parameters.

Antihistamines are inverse agonists of the histamine H_1_ receptor, competing with histamine for the normal receptor sites on effector cells of tissues such as the respiratory tract, blood vessels, gastrointestinal tract, and central nervous system (CNS).^1^, ^2^ They do not antagonize H_1_ receptors but bind to other receptor sites to shift the equilibrium of histamine receptors toward an inactive state to downregulate allergic inflammation.^1^, ^2^

There are more than 45 H_1_ antihistamines available worldwide.^2^ First‐generation antihistamines, such as triprolidine, doxylamine, and diphenhydramine, were first brought into clinical use beginning in the 1940s.^1^, ^2^ They readily cross the blood‐brain barrier (BBB) because of their liposolubility and lower molecular mass compared with second‐generation antihistamines and occupy H_1_ receptors located on postsynaptic membranes of histaminergic neurons in the CNS.^1^, ^2^, ^3^, ^4^ Poor receptor selectivity means that first‐generation antihistamines often interact with receptors of other biologically active amines, causing cholinergic, antimuscarinic, anti‐α‐adrenergic, and antiserotoninergic effects such as blurred vision, dry mouth, and gastrointestinal effects including nausea.^1^, ^4^ The main side effect associated with first‐generation antihistamines is somnolence, caused by inactivation of H_1_ receptors in the CNS, and is exacerbated by other CNS‐active substances such as alcohol.^4^ Second‐generation antihistamines, such as cetirizine, acrivastine, and loratadine, were developed from the mid‐1980s onward.^1^, ^2^ Lipophobicity and a higher molecular mass compared with first‐generation antihistamines limit their penetration of the BBB.^1^, ^2^ In addition, entry to the CNS is restricted by efflux pumps such as P‐glycoprotein located on brain endothelial cells.^5^ Moreover, they have greater specificity for H_1_ receptors and limited effect on other receptor types.^4^ Therefore, second‐generation antihistamines are associated with fewer adverse effects than first‐generation antihistamines and may benefit people managing symptoms of allergies.^1^, ^2^, ^4^

When taken at night, first‐generation antihistamines increase the latency to onset of rapid eye movement (REM) sleep and reduce the duration of REM sleep.^1^ The residual effects of poor sleep, including impaired attention, vigilance, working memory, and sensory motor performance, are often still present the next morning, depending on the antihistamine half‐life (t_1/2_).^1^ The sedating properties of the first‐generation antihistamines diphenhydramine and doxylamine are utilized in their use as over‐the‐counter (OTC) sleep aids, particularly in older adults aged older than 65 years. However, their use in this indication is considered a public health concern, as they are inappropriate when used long term or concomitantly with many common prescription drugs, and their lengthy t_1/2_ increases the likelihood of next‐day residual effects, particularly among those who are more sensitive to anticholinergic effects.^4^, ^6^

Antihistamines are used alone or in combination with other agents for the symptomatic relief of symptoms associated with the common cold or flu.^7^, ^8^ First‐generation antihistamines can reduce symptoms such as sneezing, rhinorrhea, nasal mucus, and, sometimes, cough, whereas second‐generation antihistamines offer no relief in this indication.^9^ Combinations of antihistamines with decongestants and/or analgesics are beneficial in relieving symptoms of the common cold in adults and older children compared with placebo or other active treatments; however, these benefits must be weighed against the risk of adverse effects.^8^ There is no evidence of effectiveness of antihistamine combinations for the common cold in young children.^8^

First‐generation H_1_ antihistamines became available for clinical use prior to today's requirements for clinical pharmacology, proof of efficacy through randomized controlled trials, and evidence‐based regulatory approval systems.^2^ The first‐generation antihistamine triprolidine was first developed in the 1950s and is available in combination with pseudoephedrine for the treatment of cold and allergy symptoms. Triprolidine is classified as an alkylamine, which has milder sedating effects and minimal anticholinergic activity compared with other types of first‐generation antihistamines.^10^ Animal studies have indicated that triprolidine does not have the potential for abuse, whereas diphenhydramine and chlorpheniramine demonstrated behavioral effects similar to those of psychostimulant drugs such as cocaine and amphetamines.^11^ Simons et al reported that ∼1% of triprolidine is excreted unchanged in the urine over 24 hours postadministration, suggesting that triprolidine is eliminated primarily by metabolism.^12^ First‐generation H_1_ antihistamines are metabolized by cytochrome P450 in the liver and do not function as substrates of P‐glycoprotein.^13^ Not all the metabolic routes are known; however, the majority of first‐generation H_1_ antihistamines are metabolized by (and inhibit) the CYP2D6 isozyme, which must be considered when these agents are coadministered with other drugs that are metabolized by cytochrome P450, such as metoprolol, tricyclic antidepressants, antiarrhythmic drugs, antipsychotics, and tramadol.^13^

Recent Cochrane Database systematic reviews^7^, ^8^ identified only 1 clinical trial that investigated the efficacy of triprolidine in the treatment of cold and flu. A double‐blind, randomized trial with 466 healthy adults in England evaluated the effectiveness of triprolidine alone or in combination with pseudoephedrine on cold and flu by subjective severity assessment of symptoms.^14^ There was a significantly larger reduction in the mean severity scores of sneezing and nasal obstruction in participants receiving triprolidine or triprolidine + pseudoephedrine but no significant difference in daily severity scores of rhinorrhea in participants with triprolidine ± pseudoephedrine or placebo.^7^, ^14^ Few pharmacokinetic (PK) data are available for many first‐generation antihistamines, including triprolidine. Generally, antihistamines, particularly triprolidine,^15^ are present in low concentrations in bodily fluids (∼1‐24 ng/mL; see Table 1), and although analytical methods were developed in the 1980s, the results were not widely published.^16^ Initial studies used radioimmunoassay and chromatography methods before the introduction of mass spectrometry techniques.^16^ Most first‐generation antihistamines have good oral absorption because of their liposolubility, achieving their maximum plasma concentration (C_max_) and therefore therapeutic effects within 2‐3 hours.^16^ Results of historical PK assessments of oral triprolidine are presented in Table 1.^12^, ^15^, ^17^, ^18^, ^19^, ^20^, ^21^

**Table 1:** Pharmacokinetics of Oral Triprolidine From Historical Human Studies

Author	Dose (mg)	n	Method of Detection	C_max_ (ng/mL)	T_max_ (h)	t_1/2_ (h)	AUC_0‐∞_ (ng·h/mL)
DeAngelis (1977)^18^	3.75 Syrup	16	Quantitative thin‐layer chromatography	8.2 (3.0‐17.4)	2	5 (1.5‐20)	75 (19‐182)
Miles (1990)^22^	2.5 Syrup	6	Radioimmunoassay	5.6 ± 2.9	2.0 ± 1.2	Not reported	43.3 ± 44.0
Simons (1986)^24^	0.04 mg/kg Syrup	7	High‐performance liquid chromatography	15.6 ± 8.2	1.7 ± 0.5	2.1 ± 0.8	Not reported
Findlay (1984)^21^	2.5 Tablet	3	Radioimmunoassay	3.1‐9.4	1‐2	2.3	39.0
Williams (1984)^25^	2.5 Tablet 2.5^a^ Tablet 2.5^a^ Syrup	18	Radioimmunoassay	5.5 ± 4.8 5.5 ± 5.1 6.0 ± 4.4	2.00 ± 0.86 2.06 ± 0.97 1.49 ± 0.50	2.00 ± 0.86 2.06 ± 0.97 1.49 ± 0.50	36.6 ± 46.1 50.0 ± 78.1 40.2 ± 50.2
Cohen (1985)^20^	5.0 Capsule	11	Radioimmunoassay	13.3 ± 11.1	1.91 ± 0.77	4.6 ± 4.3	132 ± 192
Perkins (1980)^23^	5.0^b^ Capsule 2.5^c^ Tablet	17	Quantitative thin‐layer chromatography	11.8 ± 1.7 13.1 ± 2.1	2.3 ± 0.3 1.6 ± 0.2	Not reported	98.0 ± 18.4 107.4 ± 24.4

AUC, area under the plasma drug concentration‐time curve; C_max_, maximum plasma concentration; h, hour; n, number of subjects; t_1/2_, elimination half‐life; T_max_, time to maximum plasma concentration.

Values indicate mean ± standard deviation, except DeAngelis (1977),^18^ which reported the range for C_max_ and no variation for T_max_, Findlay (1984),^21^ which reported the range for C_max_ and T_max_, and Perkins (1980),^23^ which reported standard error.

a Triprolidine 2.5 mg and pseudoephedrine 60 mg.

b Sustained‐action capsules (triprolidine 2.5 mg and pseudoephedrine 60 mg).

c Immediate‐release tablets (triprolidine 5 mg and pseudoephedrine 120 mg).

The aim of this study was to better characterize the PK profiles of triprolidine, given that it has properties distinct from other first‐generation antihistamines. The primary objectives of the study were to characterize the bioavailability of single‐agent triprolidine in film‐coated caplet formulations (2.5 and 5 mg) in healthy volunteers and to compare the bioavailability of these single‐agent formulations with a commercially available reference combination product (triprolidine 2.5 mg + pseudoephedrine 60 mg). The secondary objective was to assess safety. The study was completed in 2001 and sponsored by Boots Healthcare International. Reckitt Benckiser, which acquired the over‐the‐counter drugs manufacturing business of the Boots Group in 2005, is supporting the publication of the study data, having recently initiated a policy of publicly sharing data from all clinical studies.

## Methods

### Study Design

The study was a phase 1 single‐center open‐label, randomized, single‐dose, 3‐way crossover trial. Key exclusion criteria included hypersensitivity to triprolidine and history of significant disease of any body system or any condition that might interfere with the absorption, distribution, metabolism, or excretion of drugs. Full inclusion and exclusion criteria are listed in Supplemental Table 1. The study was conducted at Simbec Research Limited, Merthyr Tydfil, UK, in compliance with the ethical principles of the Declaration of Helsinki 1996 and the International Conference on Harmonization Good Clinical Practice Guidelines. All local regulatory requirements were followed, and the study was approved by the Simbec Independent Ethics Committee. The study was carried out between November and December 2001, prior to requirements to register with clinicaltrials.gov. Healthy male and female volunteers gave written informed consent and underwent screening prior to initiation of the study.

### Assessments

Clinical laboratory evaluations were conducted during screening and at completion. These included medical history and examination, electrocardiogram (ECG), hematology, clinical biochemistry, and urinalysis (tests are listed in Supplemental Table 2). Viral serology was required within 14 days prior to the study and urinary alcohol drugs of abuse screening (Syva‐EMIT d.a.u. [Syva Co., Malvern, Pennsylvania]) within 14 days prior to the study and repeated the evening before administration of each treatment. Alcohol was not allowed within 48 hours of dosing. OTC and prescription medications were prohibited in the 7 and 14 days prior to dosing, respectively, and during the study period. All adverse events (AEs) observed during the study were recorded and reported, listed and tabulated by treatment, severity, relationship to treatment, and body system according to Coding Symbols for a Thesaurus of Adverse Reaction Terms. Study procedures were performed within the Simbec Research Center, where subjects were required to stay for 2 nights during each test period. Subjects received a light meal at 7:00 pm and were required to fast from 10:00 pm the night prior to dosing. Noncaffeinated and non‐fruit‐juice liquids were allowed until 2 hours before dosing. Subjects received the 3 study medications in a crossover manner on 3 nonconsecutive dosing days with a washout period of 3‐10 days between each dose. No single‐agent formulation of triprolidine was commercially available at the time of the study, so a marketed combination product was chosen as the reference. Treatment order was allocated by a computer‐generated randomization list provided by the Clinical Trials Supplies Unit at Boots Healthcare International and approved by Nottingham Clinical Research Ltd. At the time this study was conducted, it was the policy of Boots Healthcare International to engage a third party (in this case Nottingham Clinical Research Ltd.) to generate the randomization to better protect the confidentiality of the assignment. Unblinding standard operating procedures were in place to prevent unauthorized unblinding by Reckitt Benckiser or the Clinical Research Organization (Simbec). A Latin‐square design ensured that treatment allocation was balanced for each study day. Treatment A was 1 × triprolidine 2.5 mg film‐coated caplet (test), treatment B was 1 × triprolidine 5 mg film‐coated caplet (test), and treatment C was 1 × triprolidine 2.5 mg + pseudoephedrine tablet (Actifed [McNeil Products Ltd., UK]; reference). Each dose was swallowed with 200 mL of water while in a standing position under the observation of a study investigator to confirm treatment compliance.

For PK assessments, blood samples were collected predose and 30, 45, 60, and 90 minutes and 2, 2.5, 3, 4, 6, 8, 12, and 24 hours postdose. The actual times of drug administration and blood collection were recorded, and PK analysis was performed on the relevant corrected time where necessary. Subjects remained semirecumbent for 4 hours after dosing, during which no food was allowed. Lunch and an evening meal were provided 4 and 8 hours postdose, respectively. Subjects were requested to drink 1.5 L of noncarbonated fluid during each visit to the research center. Strenuous physical exercise was prohibited during this period.

### Analysis of Plasma Triprolidine Concentration

Triprolidine was quantified (concentrations below the lower limit of quantification were substituted with zero) in plasma over the concentration range of 0.2‐20 ng/mL (nominal concentrations) by reverse‐phase liquid chromatography with tandem mass spectrometry detection (LC‐MS/MS). This procedure was developed and previously validated. Each 1 mL of plasma sample was mixed with 50 µL of 0.1 ng/µL chlorpheniramine internal standard solution in deionized water, alkalinized with 0.2 mL of 1 M sodium hydroxide, and extracted with 6 mL of dichloromethane. The upper plasma/aqueous layer was aspirated to waste, and the remaining organic solvent evaporated to dryness under a gentle stream of nitrogen at 35°C. Each sample residue was reconstituted in 50 µL of LC‐MS/MS mobile phase (ammonium formate, 0.005 M, pH 3.5 [70% v/v], acetonitrile [30% v/v]), and 20 µL was injected into the LC‐MS/MS system. An isocratic mobile‐phase flow rate of 1.2 mL/min and a split ratio of 1:7 (nominal) were employed. A Symmetry Shield RP8 HPLC column (Waters Corp., UK; 25 cm long × 0.46 cm internal diameter, 5‐µm particle size) fitted with an octadecylsilyl precolumn (Phenomenex Inc., Torrance, California; 0.4 cm length × 0.3 cm diameter) was used. Automated injection of samples was with a PE Series 200 LC pump and autosampler (Perkin Elmer, Inc., Waltham, Massachusetts), into a Sciex API 150EX atmospheric pressure ionization mass spectrometer with turbo ion spray inlet (Perkin Elmer, Inc., Waltham, Massachusetts). The following protonated molecular ions were monitored in positive ion mode: chlorpheniramine m/z 275.3, expected retention time, 3.7 minutes; triprolidine m/z 279.4, expected retention time, 4.2 minutes. Instrument control, data acquisition, and integration were with proprietary Sciex software (Perkin Elmer, Inc., Waltham, Massachusetts) running on an Apple Macintosh G3 computer system. Calibration curves were linear over plasma triprolidine concentration ranges of 0.205 and 20.446 ng/mL. Between‐run accuracy was 92.3% to 104.7%, and between‐run precision was 5.1% and 11.1%. Plasma concentrations of pseudoephedrine were not assessed in the reference product because the aim of this study was to evaluate the pharmacokinetics of single‐agent triprolidine, which was not commercially available at the time.

### Statistical Analysis

Triprolidine LC‐MS/MS quantification data were handled using Sciex MassChrom v1.1.1 incorporating MacQuan version 1.6 (Perkin Elmer, Inc., Waltham, Massachusetts). Data were analyzed using WinNonlin Pro (version 2.1; Certara USA Inc., Princeton, New Jersey), and statistics were calculated with SAS/STAT (version 6.12; SAS Institute Inc., Cary, North Carolina). Key PK parameters calculated were C_max_, time to maximum plasma concentration (T_max_), t_1/2_, area under the plasma drug concentration‐time curve up to the last measurable plasma concentration (AUC_0‐t_), and total AUC (AUC_0‐∞_). For C_max_ and AUC values, analysis of variance, including the terms for sequence, subjects nested within sequence, formulation, and period, were carried out on logarithmically transformed data and validity assessed by inspection of residual plots and the Shapiro‐Wilks test for normality. Ninety percent nonparametric confidence intervals (CIs) were constructed for the median difference in the T_max_ based on the Hodges‐Lehmann estimates. Median T_max_ was analyzed using the Wilcoxon signed rank test.

Miles et al^19^ reported a value of 0.89 and standard deviation (SD) of 0.37 for the AUC ratio for a 2.5 mg triprolidine tablet and a 5.0 mg triprolidine transdermal patch. It is likely that the AUC ratio of a tablet and a transdermal patch will be associated with greater variation than between the 2.5 mg test caplet and the combination tablet. Therefore, a value of 0.37 was considered a conservative estimate of the SD of this ratio. Assuming that the AUC ratio between the triprolidine 2.5 mg test caplet and the combination tablet would be 1, a sample size of 24 subjects would provide >80% power for 1‐sided tests to demonstrate bioequivalence, as defined by a 90%CI for the geometric mean of the AUC ratios obtained from the analysis of variance model between 80% and 125%. Bioequivalence of triprolidine between single‐agent and combination products was tested by halving (dose‐normalizing) the C_max_ and AUCs of triprolidine 5.0 mg before conducting statistical tests. Bioequivalence for AUC_0‐t_ and AUC_0‐∞_ was defined as 90%CI for the geometric mean of the ratios (the geometric mean of the relative bioavailabilities) obtained from the analysis of variance model between 80% and 125%. Triprolidine is considered to exhibit high individual variability in plasma concentration, with C_max_ reported as 13 ±11 ng/mL by Cohen et al^17^ and 15.6 ± 8.2 ng/mL by Simons, et al.^12^ Widened confidence limits of 70% to 143% have been proposed as appropriate for determining bioequivalence in drugs with high variability in plasma drug concentrations,^22^ and therefore equivalence for C_max_ was defined as 90%CI within the wider limits of 70% to 143%. These analyses of C_max_ were defined in the protocol prior to initiation of the study; current European Medicines Agency guidelines dictate that widened confidence limits may only be used when intrapatient variability of >30% is demonstrated in a study with replicate design.^23^ Both analyses were considered.

## Results

### Study Demographics

Twenty‐four subjects, 12 men and 12 women, aged 18‐50 years and weighing ≥20 to ≤27 kg/m^2^, were included in the study. The study demographics are presented in Supplementary Table 3. There was no difference in subject age between treatment groups. As expected, female subjects were significantly smaller and lighter than men, reflected in both body mass index (BMI; *P* < .01) and weight (*P* < .05). No protocol amendments or major protocol deviations were reported, there were no screening failures, and all subjects completed the study.

### Pharmacokinetics of Triprolidine

**Figure 1:**
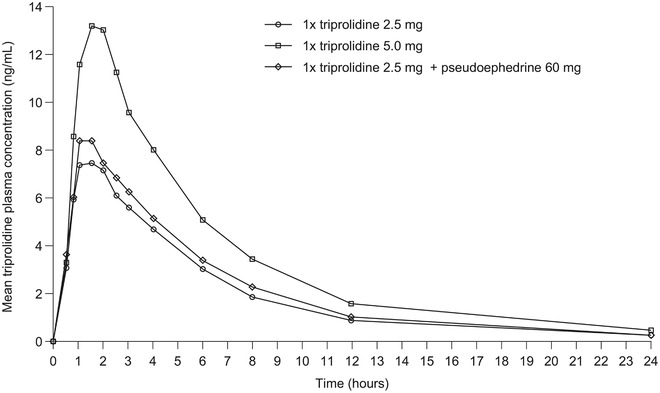
Mean triprolidine plasma concentration‐time curve. Plotted values represent arithmetic means.

The highest mean triprolidine plasma concentration for the 3 treatment arms at each time is shown in Figure 1, and all PK parameters calculated are summarized in Table 2. Plasma concentration of single‐agent triprolidine was proportional to the dose; C_max_ arithmetic means were 8.4 ng/mL for the 2.5 mg tablet and 14.3 ng/mL for the 5.0 mg tablet (7.1 ng/mL dose‐normalized). The C_max_ for 2.5 mg of triprolidine was higher when administered in combination with pseudoephedrine 60 mg (9.5 ng/mL) compared with the single‐agent formulations. The median T_max_ was approximately 1.5 hours for all 3 treatment groups. The t_1/2_ arithmetic means were comparable between groups: 3.7 hours for triprolidine 2.5 mg, 4.1 hours for triprolidine 5.0 mg (standard and dose normalized), and 4.0 hours for triprolidine 2.5 mg + pseudoephedrine 60 mg. C_max_ showed high interindividual variability in all 3 treatment groups (Supplemental Figure 1), ranging from 1.1 to 32.4 ng/mL with triprolidine 2.5 mg, from 4.6 to 31.4 ng/mL with triprolidine 5.0 mg, and from 2.2 to 21.3 ng/mL with triprolidine 2.5 mg + pseudoephedrine 60 mg.

**Table 2:** Summary of Triprolidine Pharmacokinetic Parameters

				Triprolidine 5 mg Tablet		
PK Parameter		Units	Triprolidine 2.5 mg Tablet	Actual	Dose‐Normalized	Triprolidine 2.5 mg + Pseudoephedrine 60‐mg tablet
C_max_	Arithmetic mean ± SD	ng/mL	8.4 ± 6.9	14.3 ± 6.7	7.1	9.5 ± 5.1
	Geometric mean		6.5	12.8	6.4	8.2
T_max_	Median (minimum, maximum)	H	1.5 (0.8, 3.0)	1.5 (0.8, 4.0)	1.5 (0.8, 4.0)	1.5 (0.5, 2.5)
t_1/2_	Arithmetic mean ± SD	H	3.7 ± 2.2	4.1 ± 1.6	4.1 ±1.6	4.0 ± 1.7
AUC_0‐∞_	Arithmetic mean ± SD	ng· h/mL	51.0 ± 56.1	86.0 ± 57.7	43.0	55.4 ± 35.6
	Geometric mean		35.8	68.8	34.4	45.3
AUC_0‐t_	Arithmetic mean ± SD	ng·h/mL	45.5 ± 43.5	81.1 ± 51.9	40.6	52.0 ± 33.1
	Geometric mean		32.6	65.3	32.6	42.2

AUC**_0‐t_**, area under the plasma drug concentration‐time curve up to the last measurable plasma concentration; AUC**_0‐∞_**, total AUC; C_max_, maximum plasma concentration; h, hour; SD, standard deviation; t_1/2_, elimination half‐life; T_max_, time to maximum plasma concentration.

### Relative Bioavailability of Study Medications

The geometric means for AUC_0‐∞_ and AUC_0‐t_ for each treatment group showed similar patterns; therefore, only the AUC_0‐∞_ is presented. The bioavailability of triprolidine 2.5 mg and triprolidine 5 mg was proportional with respect to the rate and extent of absorption as measured by C_max_ and AUC_0‐∞_, respectively (Table 3), with the slopes for C_max_ and AUC_0‐∞_ calculated as 0.98 and 1.00, respectively (Supplementary Table 4). Relative bioavailability of triprolidine 2.5 mg and triprolidine 5.0 mg was 101.3% (90%CI, 87.9%‐116.7%) for the ratio of C_max_ and 104.2% (90%CI, 91.0%‐119.2%) for the ratio of AUC_0‐∞_, falling within the standard confidence limits (and within the widened confidence limits for C_max_) for bioequivalence. The rate and extent of absorption were lower for triprolidine 2.5 mg and triprolidine 5.0 mg (after dose normalization) compared with triprolidine 2.5 mg + pseudoephedrine 60 mg. When comparing each of the test formulations with the reference treatment, the 90%CIs were outside the standard confidence limits of bioequivalence for both measured parameters (and also outside the widened confidence limits for C_max_). Relative bioavailability of triprolidine 2.5 mg and the reference treatment was 78.9% (90%CI, 68.4%‐90.9%) for the ratio of C_max_ and 79% (90%CI, 69.0%‐90.5%) for the ratio of AUC_0‐∞_; relative bioavailability of triprolidine 5.0 mg (after dose normalization) and the reference treatment was 77.9% (90%CI, 67.6%‐89.8%) for the ratio of C_max_ and 75.9% (90%CI, 66.3%‐86.9%) for the ratio of AUC_0‐∞_ (Table 3). There was no statistical difference in T_max_ between the reference treatment group and 2.5 mg triprolidine (*P* = .9482) or 5.0 mg triprolidine (*P* = .9492); see Table 3.

**Table 3:** Relative Bioavailability of Triprolidine

Parameter Compared	Ratio (%)	90%CI
2.5 mg triprolidine versus 5 mg triprolidine		
Ratio of C_max_	101.3	87.9‐116.7
Ratio of AUC_0‐∞_	104.2	91.0‐119.2
	*P*	90%CI
T_max_	.9482	‐0.125 to 0.500
2.5 mg triprolidine versus 2.5 mg triprolidine + pseudoephedrine 60 mg		
Ratio of C_max_	78.9	68.4‐90.9
Ratio of AUC_0‐∞_	79.0	69.0‐90.5
Ratio of AUC_0‐t_	77.4	67.7‐88.5
	*P*	90%CI
T_max_	.9482	‐0.500 to 0.000
5.0 mg triprolidine versus 2.5 mg triprolidine + pseudoephedrine 60 mg		
Ratio of C_max_	77.9	67.6‐89.8
Ratio of AUC_0‐∞_	75.9	66.3‐86.9
Ratio of AUC_0‐t_	77.4	67.7‐88.6
	*P*	90%CI
T_max_	.9492	‐0.625‐0.000

AUC**_0‐∞_**, total area under the plasma drug concentration‐time curve; AUC_0‐t_, area under the plasma drug concentration‐time curve to the last measurable plasma concentration; CI, confidence interval; C_max_, maximum plasma concentration.

### Safety

There were 42 AEs reported by 14 subjects during the study. All AEs were considered mild or moderate, except for 1 instance of severe syncope that was unrelated to study medication (triprolidine 2.5 mg). There was a clear sex difference in AE reporting with 40 of 42 AEs reported by women and the remaining 2 reported by men. Given the known pharmacology of triprolidine as a sedating antihistamine, AEs relating to sleep disturbance were expected. Mild asthenia or mild somnolence was reported 29 times in 13 subjects (11 women, 2 men). Considerable heterogeneity was observed in the temporal relationship between somnolence/asthenia and drug administration. Dosing occurred in the morning, around 9:00 am (range, 8:45 am to 9:07 am); however, the timing of somnolence/asthenia onset ranged considerably (range, 9:00 am to 6:00 pm), as did the duration (range, 1:30 am to 10:30 pm). Thirteen unexpected AEs were reported by 9 subjects (Table 4). In subjects receiving triprolidine 2.5 mg, 5 AEs were reported (2 headache, 1 sore throat, 1 dizziness, and 1 syncope), with 2 considered possibly related to treatment and 3 considered unlikely related to treatment. In subjects receiving triprolidine 5.0 mg, 3 AEs of headache were reported, with 1 considered possibly related to treatment and 2 considered unlikely related to treatment. In subjects receiving triprolidine 2.5 mg + pseudoephedrine 60 mg, 5 AEs were reported (3 of headache, 1 of loss of sensation in the left hand, and 1 of dry mouth), with 2 considered possibly related to treatment and 3 considered unlikely related to treatment. No serious AEs or deaths occurred during the study. Blood and urine analyses carried out prestudy and poststudy were compared with in‐house reference ranges. No values outside normal ranges were considered clinically significant by the investigator. All vital signs and ECG parameters measured were either within normal ranges or not considered clinically significant by the investigator.

**Table 4:** Summary of Reported Adverse Events

Adverse Event	COSTART Body System	Severity	Relationship to Study Drug	Reports, n (%)
Triprolidine 2.5 mg				
Headache	Body	Mild	Unlikely	1 (4.2)
Headache	Body	Moderate	Possible	1 (4.2)
Syncope	Cardiovascular	Severe	Unlikely	1 (4.2)
Dizziness	Nervous system	Mild	Possible	1 (4.2)
Pharyngitis	Respiratory system	Mild	Unlikely	1 (4.2)
Asthenia	Body	Mild	Possible	2 (8.3)
Asthenia	Body	Mild	Probable	1 (4.2)
Somnolence	Nervous system	Mild	Possible	2 (8.3)
Somnolence	Nervous system	Mild	Probable	4 (16.7)
Triprolidine 5.0 mg				
Headache	Body	Mild	Unlikely	2 (8.3)
Headache	Body	Mild	Possible	1 (4.2)
Asthenia	Body	Mild	Possible	2 (8.3)
Asthenia	Body	Mild	Probable	5 (20.8)
Somnolence	Nervous system	Mild	Possible	1 (4.2)
Somnolence	Nervous system	Mild	Probable	21 (8.3)
Triprolidine 2.5 mg + pseudoephedrine				
Headache	Body	Mild	Unlikely	1 (4.2)
Headache	Body	Moderate	Possible	1 (4.2)
Headache	Body	Mild	Possible	1 (4.2)
Dry mouth	Nervous system	Mild	Possible	1 (4.2)
Hypesthesia	Nervous system	Mild	Unlikely	1 (4.2)
Asthenia	Body	Mild	Unlikely	3 (12.5)
Asthenia	Body	Mild	Possible	1 (4.2)
Asthenia	Body	Mild	Probable	1 (4.2)
Somnolence	Nervous system	Mild	Possible	2 (8.3)
Somnolence	Nervous system	Mild	Probable	3 (12.5)

COSTART, Coding Symbols for a Thesaurus of Adverse Reaction Terms; n, number of subjects.

### Male‐Female Variation

Following review of safety data, an analysis of PK parameters was undertaken in male versus female subjects. C_max_ was numerically higher (*P* = .08) in women compared with men receiving triprolidine 2.5 mg (mean, 10.7 ng/mL in women versus 6.2 ng/mL in men; Table 5). T_max_ and AUC_0‐∞_ were comparable between the sexes when receiving 2.5 mg (Table 5). There were no sex differences in the PK parameters of subjects receiving triprolidine 5.0 mg or triprolidine 2.5 mg + pseudoephedrine 60 mg (Table 5).

**Table 5:** Triprolidine Pharmacokinetic Parameters by Sex

	Triprolidine 2.5 mg Tablet		Triprolidine 5.0 mg Tablet		Triprolidine 2.5 mg + Pseudoephedrine 60 mg Tablet	
Parameter	Male (n = 12)	Female (n = 12)	Male (n = 12)	Female (n = 12)	Male (n = 12)	Female (n = 12)
C_max_ (ng/mL), mean ± SD	6.2 ± 4.5	10.7 ± 8.3	14.3 ± 7.2	14.3 ± 6.5	8.7 ± 4.7	10.3 ± 5.6
	*P* = .08		*P* = .96		*P* = .49	
AUC_0‐∞_ (ng·h/mL), mean ± SD	50.2 ± 74.9	51.8 ± 31.0	82.1 ± 61.7	89.9 ± 55.7	51.0 ± 35.2	59.9 ± 37.0
	*P* = .27		*P* = .61		*P* = .49	
t_1/2_ (h), mean ± SD	3.9 ± 2.7	3.5 ± 1.5	4.0 ± 1.6	4.2 ± 1.6	4.0 ± 2.0	3.9 ± 1.3
T_max_ (h), median (range)	1.5 (1.0‐3.0)	1.5 (0.8‐2.0)	1.5 (0.8‐3.0)	1.75 (1.0‐4.0)	1.25 (0.5‐2.5)	1.50 (0.8‐2.0)

AUC**_0‐∞_**, total area under the plasma drug concentration‐time curve; C_max_, maximum plasma concentration; h, hour; SD, standard deviation; t_1/2_, elimination half‐life; T_max_, time to maximum plasma concentration.

## Discussion

The dose‐normalized PK profiles of the test triprolidine caplets (2.5 and 5.0 mg) were similar to each other and comparable to existing published data on triprolidine (Table 1).^12^, ^15^, ^17^, ^18^, ^19^, ^20^, ^21^ The PK profile of the reference triprolidine 2.5‐mg + pseudoephedrine 60 mg tablets had a greater rate and extent of absorption than the test formulations but there was no difference in T_max_ or t_1/2._ Bioequivalence between the test and reference treatments was calculated but was not the main aim of this study. Triprolidine has previously been reported as having high interpatient variability in C_max_.^12^, ^17^ This was confirmed across all 3 treatment arms in the present study, which showed up to a 30‐fold difference in the C_max_ of individual subjects receiving triprolidine 2.5 mg. DeAngelis et al^15^ suggested that absorption through the oral route is more variable than other routes of administration and that genetic and environmental factors affecting drug metabolism are involved. Individual differences that affect the absorption, distribution, metabolism, and elimination of medication affect the PK parameters for each study subject.^24^ As the present study included both male and female subjects, known differences in body composition would have likely had an effect on the range of C_max_ recorded between subjects.

Triprolidine from the test treatments was less bioavailable than the combination treatment. The reason for this is unclear; however, it may have been because of the effects of pseudoephedrine. Pseudoephedrine is a sympathomimetic drug that can affect vascular systems and gastrointestinal function and could increase the absorption of other drugs in vivo. Phenylpropanolamine, also used in cold and flu treatments, has pharmacological properties similar to pseudoephedrine and has been reported to increase the absorption of caffeine,^25^ so this may explain some of the differences in bioavailability reported in the present study. Further research is required to establish any synergies between absorption of triprolidine and concurrent administration of pseudoephedrine.

This study was not designed to provide a definitive analysis of the safety profile of triprolidine; however, the reported AE profile was as expected, none of the AEs reported impacted psychomotor activity, and AEs occurred at similar rates in the 3 treatment groups. Sex differences in the reporting of AEs are a widely accepted phenomenon,^24^ and a clear male‐female imbalance in reported AEs was observed in this study. Men and women were separated in the research facility, which may have influenced the pattern of AE reporting. Expected side effects were listed within the subject consent form, which may have influenced subjects into experiencing and/or reporting AEs. The study was not placebo‐controlled, and so subjects may have expected to experience these AEs, the so‐called nocebo effect.^26^ Despite these observations, an assessment of triprolidine PK differences between men and women was undertaken to investigate whether the differences in AEs could be because of sex‐based differences in drug clearance. These comparisons of PK parameters between male and female subjects were not appropriately powered to determine statistical significance, and findings should be interpreted with caution. Sex differences observed were numerically higher triprolidine C_max_ and AUC values in women than men for all 3 treatment groups. This may have been because of statistically significant differences in mean weight and BMI between men and women, leading to lower initial drug distribution and higher initial plasma level.^24^ However, there was no correlation between reports of expected AEs (asthenia and somnolence) and individual C_max_ values. Subjects were admitted to the test center the night before dosing, and some complained of having a bad night of sleep, which partly confounds attempts to ascribe these AEs solely to the study medication. Further research is required to identify whether sex differences in PK profiles could have any effect on the efficacy of treatment, but as differences in the present study were minimal, differences in treatment effect were not expected.

Cold and flu medications containing first‐generation antihistamines can cause side effects such as next‐day sedation, particularly in treatments with a long t_1/2_. When taken during the day, first‐generation antihistamines, even when used at the manufacturers’ recommended dose, frequently cause daytime somnolence, sedation, drowsiness, fatigue, and impaired concentration and memory. Triprolidine has a shorter t_1/2_ than other first‐generation antihistamines. In this study, the t_1/2_ of triprolidine was 3.7 ± 2.2 hours for triprolidine 2.5 mg, 4.1 ± 1.6 hours for triprolidine 5.0 mg, and 4.0 ± 1.7 hours for triprolidine 2.5 mg + pseudoephedrine 60 mg. These data are comparable to those of other PK studies evaluating oral administration of triprolidine (Table 1),^12^, ^15^, ^17^, ^18^, ^19^, ^20^, ^21^ and 1 study reported that triprolidine is undetectable in serum 12 hours after administration.^12^ In these historical studies, triprolidine was administered orally as a single agent in a syrup at 3.5,^15^ 2.5,^19^ or 0.04 mg/kg^12^; a 2.5 mg single‐agent tablet^18^, ^21^; a 5.0 mg single‐agent capsule^17^; triprolidine 2.5 mg in combination with pseudoephedrine 60 mg as a syrup^21^ or tablet^20^, ^21^; or triprolidine 5.0 mg and pseudoephedrine 120 mg in a sustained‐action capsule.^20^ By contrast, the ethanolamines^3^ doxylamine and diphenhydramine have a t_1/2_ of 10‐12 hours^27^, ^28^ and 3.4‐9.3 hours,^16^ respectively, and diphenhydramine remains detectable in serum 12 hours after oral administration.^16^, ^29^ The alkylamine^3^ chlorpheniramine has a t_1/2_ of 13.9‐43.4 hours after oral administration.^30^ Therefore, triprolidine possesses a PK profile that makes it an attractive candidate for treating symptoms of cold and flu.

## Conclusions

Results of the current study showed that the PK profile of the dose‐normalized single‐agent triprolidine formulations are equivalent and comparable to previously reported studies. Equivalence of the single‐agent and combination formulations for C_max_ and AUC_0–∞_ was not confirmed, although T_max_ and t_1/2_ were comparable in the 3 treatment arms. Expected AEs of somnolence and asthenia occurred with each treatment at a similar rate. Further studies investigating the efficacy of triprolidine as a sedating agent are needed.

## Conflicts of Interest

The authors received no financial support for the research, authorship, and/or publication of this article.

## Funding

This work was supported by Reckitt Benckiser Health.

## Author Contributions

All named authors meet the International Committee of Medical Journal Editors (ICMJE) criteria for authorship for this article. All named authors contributed to the conception and development of this article, and all authors approved the final draft and take full responsibility for the contents of the article. S.F. was the medical investigator directly responsible for the care of the trial participants; T.S. made substantial contributions to the acquisition, analysis, and interpretation of data for the work. A.S.C. made substantial contributions to the acquisition, analysis, and interpretation of data for the work. All authors contributed to critically reviewing the intellectual content of the article and have given approval to the final version.

## Supporting information


**Supplementary Figure 1**. Intersubject variability of triprolidine plasma concentration across time for (A) triprolidine 2.5 mg, (B) triprolidine 5.0 mg, and (C) triprolidine 2.5 mg + pseudoephedrine 60 mg.
**Supplementary Table 1**. Inclusion and Exclusion Criteria
**Supplementary Table 2**. Details of Blood and Urine Investigations
**Supplementary Table 3**. Study Demographics
**Supplementary Table 4**. Dose Proportionality Between Triprolidine 2.5 mg and Triprolidine 5.0 mg
