# Supplementary material for: Bioavailability of Triprolidine as a Single Agent or in Combination With Pseudoephedrine: A Randomized, Open‐Label Crossover Study in Healthy Volunteers
Source: Clin Pharmacol Drug Dev. 2020 Mar 4;9(4):486–95. doi: 10.1002/cpdd.777 (PMC7318178; doi:10.1002/cpdd.777)
Supplement: Supplementary file 1 — Supplementary Figure 1. Intersubject variability of triprolidine plasma concentration across time for (A) triprolidine 2.5 mg, (B) triprolidine 5.0 mg, and (C) triprolidine 2.5 mg + pseudoephedrine 60 mg. Supplementary Table 1. Inclusion and Exclusion Criteria Supplementary Table 2. Details of Blood and Urine Investigations Supplementary Table 3. Study Demographics Supplementary Table 4. Dose Proportionality Between Triprolidine 2.5 mg and Triprolidine 5.0 mg [file CPDD-9-486-s001.docx]

**Supplementary materials:**

**Supplementary Table 1:**

Inclusion and exclusion criteria.

| **Inclusion criteria** | **Exclusion criteria** |
| --- | --- |
| - Age: 18–50 years - Sex: Male or female - Healthy volunteer with a body mass index ≥20–≤27 kg/m^2^ - Subjects who gave written informed consent | - A history of significant disease of any body system - A history of any condition that may interfere with the absorption, distribution, metabolism or excretion of drugs - Hypersensitivity to triprolidine - A history of narrow angle glaucoma, hypertension, diabetes mellitus, hyperthyroidism, cardiovascular disease, renal and hepatic impairment, prostatic hypertrophy, stenosing peptic ulcer, pyloroduodenal obstruction or bladder neck obstruction - Myasthenia gravis or seizure disorders - A history of psychotic illness, attempted suicide or parasuicide or neurosis at any time - Current smokers and ex-smokers who have smoked within 6 months - Subjects using hypnotics, sedative anxiolytics, antidepressants, monoamine oxidase inhibitors, sympathomimetics and anti-Parkinson drugs in the previous 14 days - Ingestion of a prescribed drug at any time in the 14 days before the start of the study (excluding the contraceptive pill and hormone replacement therapy) - Ingestion of an over-the-counter preparation within 7 days before the start of the study - Ephedrine-containing dietary supplements and/or herbal stimulants based on Ephedra - A history of drug abuse (including alcohol) - Subjects with any clinically significant abnormal laboratory values - Donation of blood in quantity, e.g. to the Blood Transfusion service, or participation in another study in the past 12 weeks - Known risk factors for AIDS or known HIV positive status, or positive viral serology screen - Women of childbearing potential, who are pregnant or lactating, seeking pregnancy or failing to take adequate contraceptive precautions (i.e. an oral contraceptive, an approved hormonal implant, an intrauterine device or condoms/diaphragm and spermicide). A woman of childbearing potential is defined as any female who is less than 2 years postmenopausal or has not undergone a hysterectomy or surgical sterilization, e.g. bilateral tubal ligation, bilateral ovariectomy (oophorectomy) - Those unable, in the opinion of the investigator, to fully comply with the trial requirements - Those previously entered into the study - Those who have participated in a clinical study in the previous 3 months |

AIDS, acquired immunodeficiency syndrome; HIV, human immunodeficiency virus.

**Supplementary Table 2:**

Details of blood and urine investigations.

| **Test** | **Details** |
| --- | --- |
| Biochemistry (pre- and post-study) | Sodium, potassium, urea, creatinine, uric acid, glucose, calcium, phosphorus, total bilirubin, alkaline phosphatase, alanine transferase, aspartate transferase, gamma-glutamyltransferase, hydroxybutyrate, dehydrogenase, creatinine phosphokinase, total protein, albumin, cholesterol, triglycerides, pregnancy test (females only – at each admission for dosing) |
| Hematology (pre- and post-study) | Hemoglobin, red cells, hematocrit ratio (PCV), MCV, MCH, MCHC, white cells (differential white cell count, neutrophils, lymphocytes, monocytes, basophils, eosinophils), platelets |
| Serology (pre-study) | Hepatitis B surface antigen and C antibody, HIV antibody |
| Urinalysis (dipstick test; pre-and post-study) | pH, protein, glucose, ketones, bilirubin, blood (free hemoglobin), urobilinogen, microscopy and culture (if abnormal on routine screening), white blood cells, red blood cells |
| Drugs of abuse screen (Syva-EMIT^®^; evening before each study day) | Opiates, amphetamines, cannabinoids, cocaine, barbiturates, benzodiazepines, methadone, ethanol, MDMA (ecstasy) |

MCH, mean corpuscular hemoglobin; MCHC, mean corpuscular hemoglobin concentration; MCV, mean corpuscular volume; MDMA, 3,4-methyl​enedioxy​methamphetamine; PCV, packed cell volume.

**Supplementary Table 3:**

Study demographics.

|  | **Male** | **Female** |
| --- | --- | --- |
| **n (%)** | 12 (50) | 12 (50) |
| **Age (mean ±SD; years)** | 27.8 ±6.2 | 28.5 ±7.6 |
| **Height (mean ±SD; m)** | 1.75 ±0.08 | 1.65 ±0.04 |
| **Weight (mean ± SD; kg)** | 76.7 ±9.3 | 61.5 ±4.5 |
| **Body mass index (mean ± SD; kg/m^2^)** | 24.9 ±1.6 | 22.6 ±1.4 |

m, meter; n, number of subjects; SD, standard deviation.

**Supplementary Table 4:**

Dose proportionality between triprolidine 2.5 mg and triprolidine 5.0 mg.

| **Parameter** | **95% CI lower limit** | **Slope** | **95% CI upper limit** |
| --- | --- | --- | --- |
| **C_max_ (ng/mL)** | 0.7 | 0.98 | 1.3 |
| **AUC_0–∞_ (ng/mL/h)** | 0.8 | 1.00 | 1.2 |

AUC**_0-∞_**, total area under the plasma drug concentration/time curve; CI, confidence interval; C_max_, maximum plasma concentration.

**Supplementary Figure 1:**

Inter-subject variability of triprolidine plasma concentration across time for A) triprolidine 2.5 mg, B) triprolidine 5.0 mg, and C) triprolidine 2.5 mg + pseudoephedrine 60 mg.


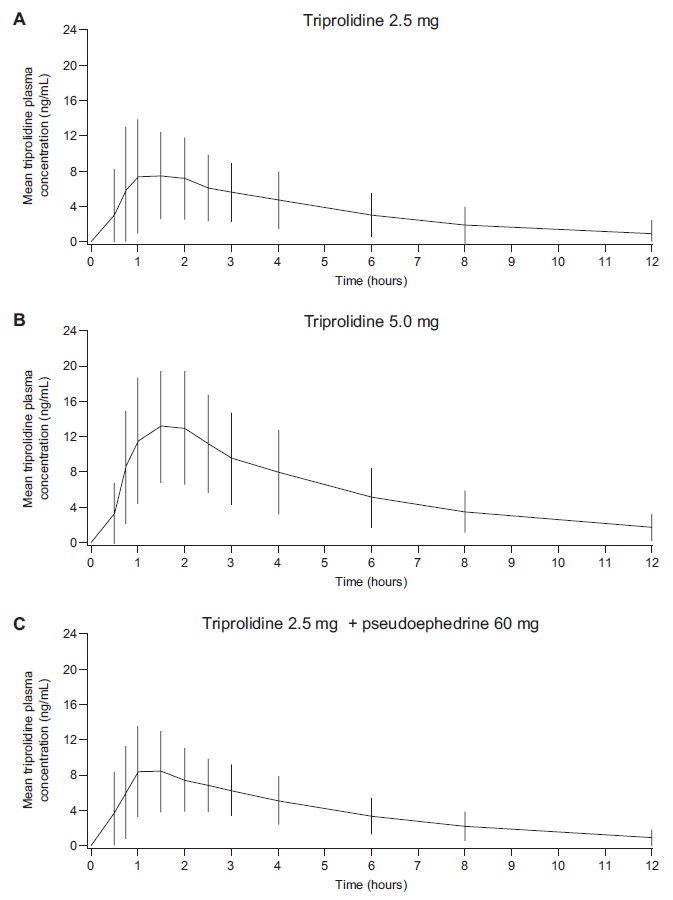


Plotted values represent arithmetic means ± standard deviation
